# Longitudinal Changes and Predictive Value of Multiparametric MRI Features for Prostate Cancer Patients Treated with MRI-Guided Lattice Extreme Ablative Dose (LEAD) Boost Radiotherapy

**DOI:** 10.3390/cancers14184475

**Published:** 2022-09-15

**Authors:** Ahmad Algohary, Mohammad Alhusseini, Adrian L. Breto, Deukwoo Kwon, Isaac R. Xu, Sandra M. Gaston, Patricia Castillo, Sanoj Punnen, Benjamin Spieler, Matthew C. Abramowitz, Alan Dal Pra, Oleksandr N. Kryvenko, Alan Pollack, Radka Stoyanova

**Affiliations:** 1 Department of Radiation Oncology, University of Miami Miller School of Medicine, Miami, FL 33136, USA; 2 Biostatistics and Bioinformatics Shared Resource, Sylvester Comprehensive Cancer Center, University of Miami Miller School of Medicine, Miami, FL 33136, USA; 3 Department of Public Health Sciences, University of Miami Miller School of Medicine, Miami, FL 33136, USA; 4 Sylvester Comprehensive Cancer Center, University of Miami Miller School of Medicine, Miami, FL 33136, USA; 5 Department of Radiology, University of Miami Miller School of Medicine, Miami, FL 33136, USA; 6 Desai Sethi Urology Institute, University of Miami Miller School of Medicine, Miami, FL 33136, USA; 7 Department of Pathology and Laboratory Medicine, University of Miami Miller School of Medicine, Miami, FL 33136, USA

**Keywords:** prostate cancer, radiotherapy, multiparametric MRI, radiomics, imaging biomarkers, machine learning, dynamic contrast enhancement, peritumoral features

## Abstract

**Simple Summary:**

In this study, we investigated the longitudinal changes and predictive value of multiparametric MRI (mpMRI) features of prostate cancer patients receiving Lattice Extreme Ablative Dose (LEAD) boost radiotherapy (RT). Ninety-five mpMRI from 25 patients, acquired pre-RT and at 3 time points following RT were analyzed. Five regions of interest were analyzed, related to tumor, peritumoral, and normally-appearing tissues. We identified significant changes in the imaging parameters following RT in all regions. By using selected features at the four scan points and their differences (Δ radiomics), we were able to build viable predictive models for endpoint biopsy positivity. Our study demonstrates that RT causes significant changes to quantitative imaging features in both tumorous and normally-appearing tissues. Because of the noninvasive nature of the mpMRI, acquiring multiple exams post-RT is feasible to monitor treatment response. Several quantitative imaging features are promising predictors of treatment failure based on post-treatment positive biopsy, a strong marker for clinically relevant endpoints.

**Abstract:**

We investigated the longitudinal changes in multiparametric MRI (mpMRI) (T2-weighted, Apparent Diffusion Coefficient (ADC), and Dynamic Contrast Enhanced (DCE-)MRI) of prostate cancer patients receiving Lattice Extreme Ablative Dose (LEAD) radiotherapy (RT) and the capability of their imaging features to predict RT outcome based on endpoint biopsies. Ninety-five mpMRI exams from 25 patients, acquired pre-RT and at 3-, 9-, and 24-months post-RT were analyzed. MRI/Ultrasound-fused biopsies were acquired pre- and at two-years post-RT (endpoint). Five regions of interest (ROIs) were analyzed: Gross tumor volume (GTV), normally-appearing tissue (NAT) and peritumoral volume in both peripheral (PZ) and transition (TZ) zones. Diffusion and perfusion radiomics features were extracted from mpMRI and compared before and after RT using two-tailed Student t-tests. Selected features at the four scan points and their differences (Δ radiomics) were used in multivariate logistic regression models to predict the endpoint biopsy positivity. Baseline ADC values were significantly different between GTV, NAT-PZ, and NAT-TZ (*p*-values < 0.005). Pharmaco-kinetic features changed significantly in the GTV at 3-month post-RT compared to baseline. Several radiomics features at baseline and three-months post-RT were significantly associated with endpoint biopsy positivity and were used to build models with high predictive power of this endpoint (AUC = 0.98 and 0.89, respectively). Our study characterized the RT-induced changes in perfusion and diffusion. Quantitative imaging features from mpMRI show promise as being predictive of endpoint biopsy positivity.

## 1. Introduction

Prostate cancer has a long natural history [1], and local persistence after primary treatment can remain clinically undetectable for over 10 years [2,3]. Common definitions of treatment failure after definitive prostate cancer radiotherapy (RT), such as the Phoenix criteria [4], use longitudinal PSA measurements to assess persistence or recurrence of disease. However, 2- to 3-years after completion of definitive RT, residual prostate cancer cells can be seen on biopsy in 40–50% of men who otherwise appear disease-free [5,6]. Post-treatment positive biopsies are strong predictors for biochemical progression, metastatic disease, and prostate cancer-specific mortality [7,8,9,10,11,12]. Prostate regions, with the greatest tumor burden and grade, are at the highest risk of harboring persistent disease after RT [13,14,15]. An alternative, non-invasive biomarker able to longitudinally interrogate high-risk regions of the prostate after definitive RT could provide great clinical utility. Quantitative imaging features derived from multiparametric MRI (mpMRI) play an undisputed role in discriminating between tumor and normally-appearing prostate tissue (NAT) and are good candidates to fill this space. However, very few studies have investigated their role as early surrogate biomarkers of RT response [16]. Similarly, RT-induced tissue changes in prostate cancer and normal prostate tissue identified in serial mpMRI of the prostate before and after RT have not been thoroughly investigated.

Diffusion-weighted imaging (DWI) and dynamic contrast-enhanced (DCE) sequences in mpMRI provide quantitative measurements related to functional properties of tissues [17,18]. DWI evaluates the Brownian motion of water molecules, which is restricted in cancer-harboring tissues. Water diffusion in the prostate is influenced by tissue composition, including cellularity, stromal content, and vascularization. Diffusion properties are characterized by the apparent diffusion coefficient (ADC). DCE-MRI evaluates the vascularity of the prostate in order to identify permeability changes related to tumor angiogenesis and is sensitive to differences in prostate cancer micro-vessel architecture [19,20]. DCE-MRI series are obtained before, during, and after injection of gadolinium-based contrast agents. Most pharmacologic models used for quantitative analysis determine the rate of contrast exchange between blood plasma and extracellular space with transfer rate constants such as K^trans^-forward volume transfer constant and k_ep_-reverse reflux rate constant between extracellular-extravascular space (EES) and plasma. Another estimated parameter is v_e_, which represents the EES fraction [21,22]. In summary, DWI, and DCE-MRI sequences of mpMRI result in quantitative features that have direct physiological interpretation.

In this study of prospectively collected data, we characterize longitudinal changes in tumor, peritumoral, and normally-appearing tissues of the peripheral (NAT-PZ) and transition zones (NAT-TZ) following administration of Lattice Extreme Ablative Dose (LEAD) RT. The LEAD technique, a spatially-fractionated external beam RT (EBRT) conceptualized as a depth extension of GRID RT [23,24] is a novel treatment designed to target high-risk regions of prostate cancer by delivering dose-escalated RT without increasing toxicity to nearby normal tissues [25]. Longitudinal mpMRI data were acquired pre-RT and at three time points post-RT from patients enrolled in two contemporary clinical trials using the LEAD treatment. Quantitative imaging features associated with the functional mpMRI sequences are summarized. We also investigate the ability of these features to predict treatment response based on two-years post-treatment biopsies.

## 2. Materials and Methods

### 2.1. Patients

Prostate cancer patients received the LEAD RT as part of one of the University of Miami investigator-initiated clinical trials: The Miami BLaStM trial (ClinicalTrials.gov: NCT02307058) or the LEAD trial (ClinicalTrials.gov: NCT01411319). Both trials were approved by the Institutional Review Board at the University of Miami. Written informed consent was obtained from all patients. All biopsies were reviewed by an experienced GU specialist pathologist (ONK) at the University of Miami according to the most contemporary prostate cancer grading recommendations [26]. For diagnostic biopsies performed at outside referring institutions, the biopsy slides were submitted for central assessment at the University of Miami. Each individual biopsy core was assessed separately. Case grade was assigned based on the biopsy core with the highest grade of cancer [27]. Intraductal carcinoma of the prostate was not graded [28,29].

The LEAD RT technique was described in detail by Pollack et al. [25]. Briefly, it involved placing 1–3 dose cylinder(s) in mpMRI-defined Gross Tumor Volumes (GTVs), wherein each dose cylinder was treated to 12–14 Gy on day 1. Standard fractionation of the planning target volumes (PTVs) was started on day 2 with treatment of the prostate and proximal seminal vesicles (SVs) at 2.0 Gy per day for 38 fractions to 76 Gy. MRI/Ultrasound (MRI/US)-fused targeted biopsies were acquired pre- and at 2–2.5 years post-RT (endpoint biopsy).

### 2.2. MpMRI Acquisition

**Figure 1:**
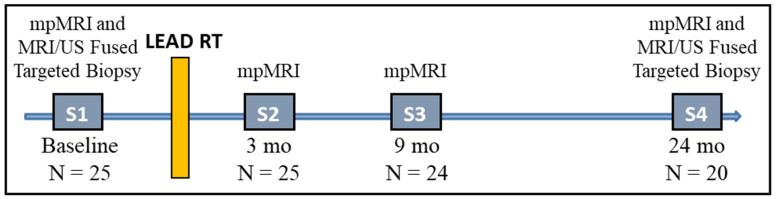
Timeline of mpMRI and biopsy acquisition. At each time point the number of analyzed patients is shown. Post-RT series were acquired at 3-, 9-, and 24-months after end of treatment.

mpMRI exams were performed pre-RT (baseline) and at 3-, 9-, and 24-months post-RT. Figure 1 illustrates the timeline of longitudinal mpMRI and biopsy acquisition.

**Figure 2:**
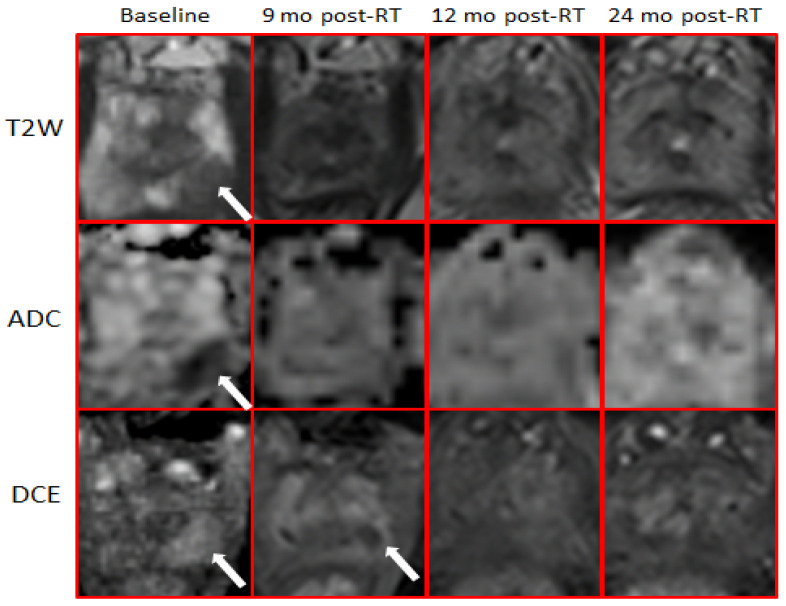
Longitudinal changes of mpMRI following RT. T2-weighted, ADC, and early enhancing images from the DCE-MRI series are shown. At baseline, the arrows indicate the GTV, defined by hypointensity on T2-weighted, ADC, and enhancing on DCE. There is no hyperintensities on T2-weighted and ADC post-RT. DCE at 3-months post-RT indicates complete obliteration of the GTV (white arrow).

All patients in the study received mpMRI with sequences and sequence parameters consistent with the recommendation for PIRADS v2 [30]: T2-weighted (T2W) MRI, T1-weighted (T1W), DCE-MRI, and DWI with the generation of ADC maps. Patients were asked to have a moderately full bladder and empty rectum. mpMRI data was acquired using 3T Discovery MR750 (GE, Waukesha, WI, USA), 3T MR Magnetom Trio, Skyra and 1.5T Symphony (Siemens, Erlagen, Germany). Acquisition parameters of the individual sequences on Discovery MR750 and Skyra are listed in Appendix A Table A1. Figure 2 illustrates the representative axial slices from the T2W, DCE (early enhancing image) and ADC shown at the four scan points of mpMRI acquisition.

### 2.3. Endpoint Biopsy

At 2–2.5 years after completion of RT treatment, the patients underwent a 12-core template needle biopsy of the prostate, and 2 additional biopsy cores were obtained from any suspicious functional mpMRI areas and/or the original site of biopsy confirmation of prostate cancer at diagnosis. The rationale for the biopsies at 2–2.5 years was to evaluate the extent of disease eradication, as well as the prognostic significance of positive biopsies in men who were otherwise free of evidence of disease (no evidence of biochemical and/or clinical failure).

### 2.4. Prostate and GTVs Segmentations

The prostate and prostate peripheral zone (PZ) were manually contoured on the diagnostic (pre-RT) T2W in MIM software (MIM Software Inc., Cleveland, OH, USA). The GTVs were determined based on mpMRI-defined suspicious lesions recognized by early phase contrast enhancement on DCE-MRI or by water restriction on the ADC, along with abnormalities on T2W imaging [25]. When necessary, in case of patient or other movements, the ADC and/or DCE-MRI were co-registered to T2W, using the fusion utilities in MIM software. Prostate, PZ, and GTV contours were transferred onto 3-, 9-, and 24-months post-RT mpMRIs. In all cases, prostate and PZ contours were adjusted to account for prostate size changes following RT. GTVs were not changed, and all efforts were made to position the GTV contour over the area of the RT boost. In this process, anatomical landmarks, such as the urethra or ejaculatory ducts, were also used as references.

### 2.5. Radiomic Features Extraction

For each mpMRI exam (Figure 1), axial T2W, ADC maps, and DCE images were resampled and co-registered to a uniform pixel spacing of 0.5 × 0.5 mm^2^. Using the prostate contours, they were cropped to the prostate region of interest (with 10 pixels (5 mm) padding along the x and y coordinates). Similarly, all volumes were interpolated to 3 mm slice thickness to account for resolution differences during acquisition.

**Figure 3:**
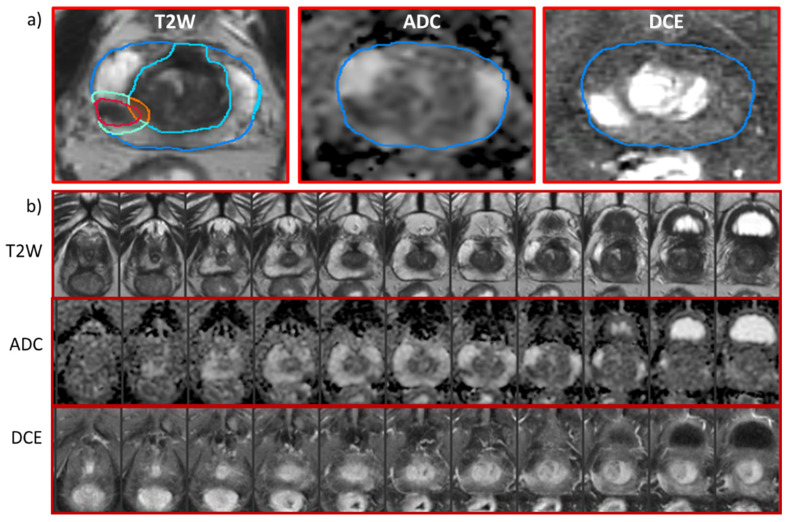
MpMRI sequences and segmented ROIs for analysis. (**a**) Representative axial slices from mpMRI of a patient at a baseline: T2-weighted, ADC, and early enhancing DCE-MRI series. The contours in the T2-weighted image are of the prostate (blue) and PZ (cyan). Gross Tumor Volume (GTV) is outlined in red; peri-tumoral GTV is represented by turquoise within the peripheral zone (PT-PZ) and orange contour within transitional zone (PT-TZ). (**b**) Axial slices through the entire prostate from apex to base on T2-weighted, ADC and early enhancing DCE-MRI series. The eight slice (from left to right) corresponds to the image in panel a).

The contours of the transition zone (TZ) were calculated by subtracting the PZ from the prostate regions. To capture the immediate GTV environment, peritumoral (PT) rings of 5 mm thicknesses were automatically defined (PT-GTV) [31]. The intersections of the PT-GTV with PZ were assigned as PT-PZ and, correspondingly, the intersections with TZ were assigned as PT-TZ. NATs in the PZ and TZ (NAT-PZ and NAT-TZ) were calculated as the areas of PZ and TZ that are outside of the PT-GTV. The contours considered in the study are shown in Figure 3.

Eleven first-order statistics were calculated for each ROI on ADC. These features included histogram characteristics such as percentiles, mean, standard deviation, skewness, and kurtosis [32]. For DCE analysis, the ‘Extended’ Tofts Model [21,22] was applied to the averaged contrast-to-time curve from each ROI. Using synthetic Parker fixed population average Arterial Input Function (AIF) [33], a valid compartmental modeling was carried out even at the lower temporal resolution of the data [34]. The determined features were: K^trans^ (min^−1^), a volume transfer constant of the contrast related to perfusion and permeability per unit volume of tissue; k_ep_ (min^−1^), the transport rate constant of the contrast from the extracellular-extravascular space (EES) to the vascular space; and v_e_, the EES fraction. In addition, t_onset_, the time of contrast wash-in; AUC90 and AUC120, the areas under the averaged contrast-to-time curve for the first 90 and 120 s, respectively, were calculated. A summary of the considered ROIs and radiomic features is provided in Table 1. The radiomics pipeline was implemented in MATLAB (MathWorks Inc., Natick, MA, USA) and were calculated within the annotation regions for each MRI protocol independently.

### 2.6. Modeling and Statistical Analysis

The ADCs and DCE features at baseline were compared between the GTV, NAT-PZ, and NAT-TZ using two-tailed Student t-tests. The same tests were used to describe the changes longitudinally pre- and post-RT. The features at the four mpMRI scan points (S1–S4) and their differences were investigated for correlation with the endpoint biopsy. Ten comparisons were conducted with the dichotomized outcome variable. For each comparison, the radiomics features were assessed for association with the endpoint biopsy positivity via a univariate logistic regression. Features that are significant (*p*-value < 0.05) were then ranked based on ascending Welch’s t-test *p*-value, and the first (lowest *p*-value) feature is selected. An iterative procedure was devised to select an uncorrelated subset of the remaining features by removing features that are highly correlated (Pearson r > 0.85) with an already selected feature. The set of selected features are modeled using a multivariate logistic regression. The AUC for the model is reported as a performance measure and is calculated using the Leave-One-Out method. The model performance was also summarized in the confusion matrix.

## 3. Results

MpMRI from 25 patients, acquired between 2011 and 2018, who received the LEAD treatment, were analyzed. The cohort consisted of all patients from the LEAD trial (n = 21) and 4 patients with positive endpoint biopsy from the BlaStM trial. Baseline clinical, pathologic, and other characteristics are reported in Table 2. The median age of the patients was 68 years (range 44–85). Clinical T-categories were T1c in 64%, T2a in 20%, T2b in 12%, and T2c in 4%. The median pre-treatment PSA was 6.3 ng/mL (range 3.34–15.91). The median time between the baseline mpMRI and RT start was 58 days, range 27–139; the median time between MRI/US biopsy and RT start was 21 days, range 13–50.

**Figure 4:**
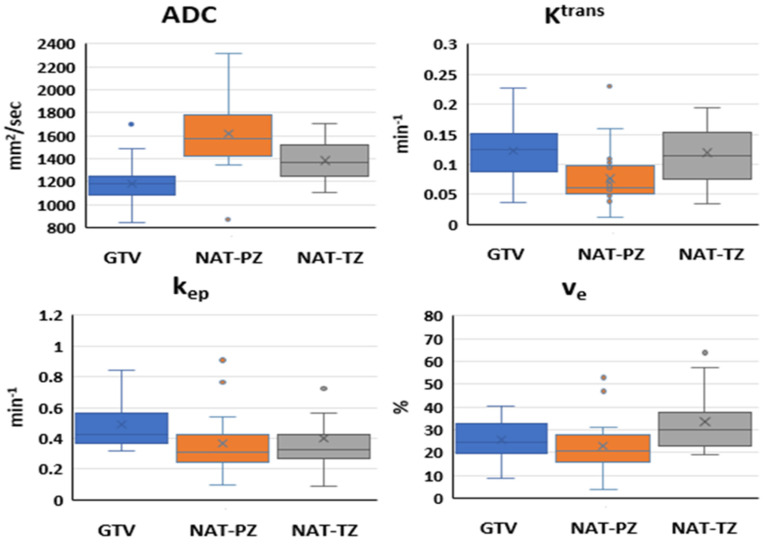
Quantitative imaging features in analyzed ROIs at baseline: Box and whisker plots comparing ADC, K^trans^, k_ep_, and v_e_ in GTV, NAT-PZ, and NAT-TZ. ADC values were significantly different between the three ROIs. K^trans^ in NAT-PZ was significantly lower than in the other two ROIs. k_ep_ in GTV was significantly higher than in the other two ROIs. v_e_ in NAT-TZ was significantly higher than in other two ROIs (*p* = 0.001).

In total, 94 mpMRI examinations from 25 patients, including pre-RT (N = 25), 3 months post-RT (N = 25), 9 months post-RT (N = 24), and 24 months post-RT (N = 20) (Figure 1) were analyzed. The ADC and the DCE pharmaco-kinetic parameters from the baseline mpMRI exams are shown in Figure 4 where the box plots represent the average values (Table 3) of the features in three volumes: GTV, NAT-PZ, and NAT-TZ. The baseline ADC values were significantly different between the three volumes (Appendix A Table A2), with the GTV having the lowest ADC (mean ± stdev: 1177.84 ± 217.12), followed by NAT-TZ (1381.76 ± 170.34) and NAT-PZ (1616.37 ± 304.02). K^trans^ was significantly lower in NAT-PZ in comparison with the other two volumes. k_ep_ was significantly different between GTV and NAT-TZ; k_ep_ in NAT-TZ was significantly higher than in other two ROIs.

**Figure 5:**
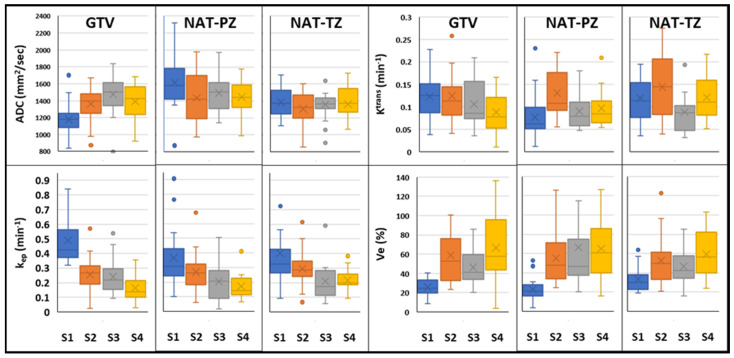
Longitudinal changes of quantitative imaging characteristics before and after LEAD-RT: Box and whisker plots comparing ADC, K^trans^, k_ep_, and v_e_ in GTV, NAT-PZ, and NAT-TZ from longitudinal mpMRI exams acquired at pre-RT (S1) and at 3 scans at 3- (S2), 9- (S3), and 24-months post-RT (S4).

The longitudinal changes of the diffusion and perfusion imaging features before and after LEAD-RT are shown in Figure 5, where the box plots represent the average values (Table 3) of the features in three volumes: GTV, NAT-PZ, and NAT-TZ. ADC, k_ep,_ and v_e_ changed significantly in the GTV at 3 months post-RT in comparison with baseline (Appendix A Table A3). The 3-months post-RT ADC changes in GTV and NAT were in opposite directions; ADC increased in the GTV (15.67%) and decreased in the NAT by 11.19% in PZ and 5.63% in TZ. ADC increased in all volumes in the 9-months exam with the most pronounced change in GTV (25.21% relative to the baseline and 8.24% relative to 3 months). ADC values levelled off at 24 months and while the ADC values at baseline were statistically different between the three volumes (Figure 5), there was no significant difference at the last measurement. In contrast, k_ep_ continued to significantly decrease (*p* < 0.001) gradually in GTV (47.89%, 50/63%, and 66.43% relative to baseline). The most dramatic change in K^trans^ was in the NAT (−69.79% in PZ and +20.80% in TZ). Similarly, to ADC, in all perfusion comparisons, the values in the 3 volumes at 24 months were not statistically different.

**Figure 6:**
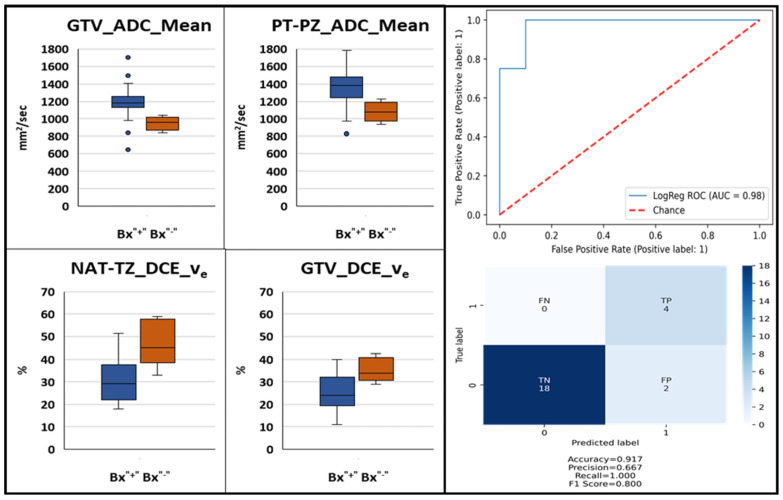
Baseline mpMRI-based model for the prediction of second year post-RT biopsy positivity. Box and whisker plots identified significantly different (*p*-value < 0.05) quantitative ADC and DCE imaging features between patients with negative and positive second year biopsy (**left**). Receiver operating characteristic curve of a logistic regression model built using these baseline features and confusion matrix (**right**).

**Figure A1:**
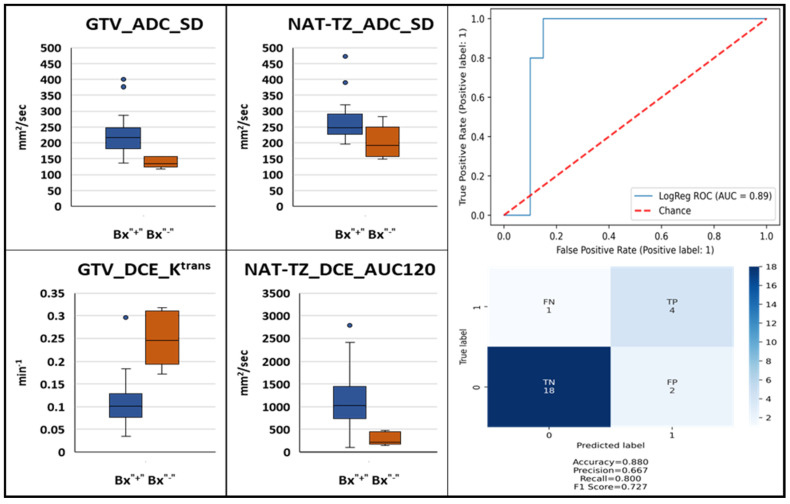
Three-months post-RT mpMRI-based model for the prediction of second year post-RT biopsy positivity. Box and whisker plots of identified significantly different (*p*-value < 0.05) quantitative ADC and DCE imaging features between patients with negative and positive second year biopsy (**left**). Receiver operating characteristic curve of a logistic regression model built using these baseline features and confusion matrix (**right**).

Using the procedure described in the Methods sections, the associations of the imaging features at each of the four scan points (Figure 1) with the second-year biopsy positivity was investigated. Five of the patients had positive biopsies after two years. One of these patients was excluded from the baseline model because the acquired DWI was of low quality. Four features were identified to be significantly associated at the baseline and 3 months post-RT and none at 9- and 24-months mpMRIs. The features, selected for the baseline radiomics logistic regression model, were: GTV_ADC_Mean, GTV_DCE_v_e_, PT-PZ_ADC_Mean, and NAT-TZ_DCE_v_e_ (of a note, both GTV_ADC_Mean and GTV_ADC_90% were significant). The Receiver Operating Characteristic (ROC) curve for the baseline model and the confusion matrix are shown in Figure 6. The baseline model shows high predictive power of endpoint biopsy positivity (AUC = 0.98, F_1_ Score = 0.80). The confusion matrix indicates that all four patients with positive biopsies were correctly classified. For the three-months post-RT model, the selected features were: NAT-TZ_DCE_AUC120; GTV_DCE_SD, GTV_DCE_K^trans^, and NAT-TZ_ADC_SD. This model also shows high predictive power of endpoint biopsy positivity (AUC = 0.89, F_1_ Score = 0.727). The ROC curve for the three-months post-RT model and the confusion matrix is shown in Figure A1.

## 4. Discussion

Early biomarkers for RT response are of paramount importance, especially for prostate cancer, which requires years of follow-up to determine treatment failure. In this work, we chose to characterize the longitudinal changes in several quantitative imaging features that are related to the physiological and functional response to therapy. ADC, a marker of tissue water diffusion restriction and cellularity, K^trans^, and other features related to vascular perfusion/permeability, could provide useful quantitative, physiological, and functional information about the effects of radiotherapy on prostate cancer patients. To the best of our knowledge, this is the first study to combine quantitative diffusion and perfusion at multiple scan points (pre- and post-RT) to monitor the changes not only in the tumor (GTV) but also in peritumoral and normal tissues. We also built a radiomics-based prediction model for patient outcome using the second year post-RT biopsy as an endpoint.

The expression of the features at baseline in GTV and NAT is consistent with the tissue composition of the tumor and normal PZ and TZ. Low ADC values in GTV (Figure 4) reflect the greater epithelial composition of the prostate cancers, the majority of which are epithelial in nature. Prostate epithelium had lower ADC values than stromal and luminal space [35]. Histologically, the prostate is made up of multiple glands, each with acini that produce secretions that are released into the seminal fluid. In contrast, the prostate stromal tissue is fibromuscular with smooth muscle admixed with fibroblasts. In average, about 70% of the PZ is comprised mainly by glands while TZ has predominantly a stromal composition. High ADC values in the NAT-PZ (Figure 4) resulted from the prostatic-fluid in the lumen of the glands of benign treatment-naïve prostate tissue.

K^trans^ reflects the uptake of the contrast agent, which is affected by the combined effect of tissue blood flow and permeability. The high K^trans^ at baseline in GTV was due to the increased neovascularization, vascular density, and vascular permeability in the tumor [36,37]. NAT-PZ is characterized by low K^trans^ due to the predominantly glandular composition of the PZ. The basement membrane lining the benign glands does not allow the contrast to penetrate inside the glands [38]. k_ep_ is a parameter that reflects the return of the contrast agent to the blood vessel. The increased neovascularization of the tumor may have higher vascular density per unit area and altered vascular structure that may lead to an increased permeability, making it easier for the contrast to return back to the vascular space, explaining a significantly higher k_ep_ in GTV.

The observed ADC’s increase in GTV following RT is consistent with the significant reduction of the tumor volume and reduced tumor cellularity, the dispersed individual cell pattern of cancer cell distribution. Conversely, ADC is decreased in NAT-PZ. These findings align well with the fact that RT is known to induce involution of glandular tissue in addition to increased stromal volume on histopathology. In summary, during the early stages after RT, the GTV, NAT-PZ, and NAT-TZ have different dynamics of imaging feature characteristics, but after two years the radiological appearance becomes similar between the three compartments. Such a phenomenon is most likely explained by the natural evolution in cancer and benign tissue of different zones of the prostate that takes place over time after irradiation.

The increase of tumor ADC values after radiation therapy is reported in several studies. Pasquier et al. report median ADC (n = 13) at baseline 1140 mm^2^/s and at 6- and 12-month post-RT- 1160 mm^2^/s and 1520 mm^2^/s, respectively [39]. Foltz et al. found an increase of ADC values from 1130 mm^2^/s to 1300 mm^2^/s in the tumor during EBRT, where MRI was acquired every two weeks throughout eight weeks of image-guided prostate IMRT (n = 17) [40].

Radiation causes devascularization in both tumorous and benign prostatic tissue [37]. This aligns with our finding of a significant overall decrease in the K^trans^ in GTV. In Franiel et al., the initial increase in K^trans^ in NAT was attributed to the inflammatory reaction of the tissue to radiotherapy [41].

This study is the first to comprehensively evaluate pre- and post-RT mpMRI associations with a second-year biopsy positivity endpoint. Prostate biopsies taken at 2–3 years after RT are strongly associated with eventual biochemical failure, clinical failure, and survival [7,11,42]. Biopsy data from our EBRT experience and that of others indicates that residual prostate cancer cells at 2–3 years post-RT may be seen in up to 40–50% of men who otherwise appear to be disease-free [7,8]. About 50% considered biopsy positive experience biochemical failure (BF) 8–10 years later, while biopsy negative experienced about 20% BF [11,43]. Data from the Randomized Hypofractionation Trial [44,45] confirmed that post-Tx prostate biopsy is the strongest predictor of patient outcome. Akin et al. [46] and later the same team in the study by Donati et al. [47] investigated radiologists’ evaluation of post-RT mpMRI in detecting locally recurrent prostate cancer in patients in whom recurrence was clinically suspected (BF defined by the “Phoenix” criteria (PSA nadir plus 2 ng/mL) [4], or consecutively rising PSA yet still insufficient to fulfill the “Phoenix” criteria). The endpoint in these studies was also post-RT biopsies but, unlike in our study, the biopsies were collected on average at a later time point (approximately 4-years post-RT). The authors also reported differences in ADC and K^trans^ for the patients with positive and negative biopsies. Of a note, mpMRI in these studies was acquired shortly after the post-treatment biopsy. In contrast, our study identified imaging features that potentially can serve as actionable biomarker at early scan points.

Several quantitative imaging features are promising predictors of treatment failure based on post-treatment positive biopsy, a strong marker for clinically relevant endpoints. About a third of the investigated patients in a randomized hypofractionation trial had positive biopsies [48]. In addition, the radiation boost decreases the odds of post-treatment positive biopsy [49] and in the LEAD trial there was only one patient with positive biopsy. We enriched the analyzed dataset for failures by adding four patients with positive endpoint biopsy from the BlaStM trial. The power of mpMRI features in predicting RT outcome using PSA-based endpoints has been investigated before. Chatterjee et al. identified a strong association of ADC values with BF, defined using the Phoenix criteria [16]. Using mpMRI from 51 patients (7 failures) with a median follow-up of 65 months, an ADC cutoff of 960 mm^2^/s was determined with a sensitivity of 100% and a specificity of 48% for predicting biochemical failure. Although the authors caution that the absolute ADC values might vary slightly between scanners and with the choice of imaging parameters, this cutoff point clearly separated the two groups of patients in our study (Figure 6, first panel). In fact, when we tested the 960 mm^2^/s cutoff point in our data at baseline, we correctly classified 3 out of the 4 patients with positive second year biopsies. It is apparent that the strong prognostic signal in the ADC values drives the high performance of our baseline model (AUC = 0.98). Interestingly, when DCE features were investigated, Chatterjee et al. did not identify any associations of these features with failure [16]. Using biochemical recurrence (BCR) after IMRT, Yamaguchi et al. identified significant association of the ADC ratio of tumor to normal prostate tissue analyzing DWI from 101 patients (10 failures) with a median follow up of 29 months [50]. In multivariate analysis, they showed that a cutoff point of 0.59 was associated with a significantly higher rate of BCR. Assuming that the representative region in normal tissue is selected in the PZ, our results are in agreement with this report. ADC values, before and after RT, were also investigated in a report by Lui et al. [51]. Seventy-eight high-risk prostate cancer patients were included in the analysis. Thirteen patients suffered recurrence within 3 years (12 with biopsy-proved local recurrence and 1 with BCR) and 65 patients were recurrence free for over 3 years. Post-RT exams were acquired at an average of 8.7 weeks (range: 5–17 weeks) following RT. The authors identified significant differences in ADC between the two groups of patients on the post-RT exam while no significant differences were found at baseline. ROC analysis revealed that post-IMRT ADC values could help identifying patients suffering recurrences (AUC, 0.88; *p* < 0.001) with a cut-off value for the post-IMRT ADC value of 1340 mm^2^/s (sensitivity, 69.2%; specificity, 89.2%).

Studying the peritumoral tissue used for investigating imaging changes post-RT is a novel element in our study. Peritumoral regions may encode useful information as prostate cancer influences its surrounding habitat [31]. This additional information has significantly improved the capability of radiomics in risk stratification. Incorporating peritumoral regions while extracting radiomic features complemented the intra-tumoral information. It also improved their predictive power when utilized individually or while training machine learning models. Indeed, one out of the four imaging features in the baseline-prediction model (Figure 6) was a peritumoral feature.

Risk stratification is critical for determining the optimal treatment for patients with localized prostate cancer. mpMRI-derived quantitative features are appealing as imaging biomarkers because the acquisition is noninvasive and does not use ionizing radiation, yet it is quantitative. DWI-derived features are especially attractive as they do not require any exogenous contrast agents and can be obtained relatively rapidly [52]. By detecting RT failures earlier, patients could be selected for treatment intensification with the goal of preventing distant metastasis and improving overall survival.

There are several limitations to consider for this analysis. The preliminary findings of this pilot study should be validated in a larger prospective cohort. The research was conducted within a single-institution academic setting with subspecialized multidisciplinary expertise and the performance of the created models may not be generalizable to community practice. The performance of the models will most likely deteriorate in larger, more heterogenous dataset. The schema of the clinical trial included 4 mpMRIs exams. MpMRI exams are expensive and often cumbersome for the patients. One of the aims of a future study with a larger cohort is to identify the optimal number and time-point of collection after RT to mpMRI with the goal to reduce the number of mpMRIs.

This work aimed at exploring the prognostic value of quantitative mpMRI features as a potential clinical tool to identify which patients would fail curative radiotherapy. As such, many questions for clinical post-radiotherapy management selection are beyond the scope of this work.

We limited the analysis to perfusion and diffusion sequences of the mpMRI because we were interested in changes with direct physiological interpretation. For the same reason we selected the pharmaco-kinetics parameters for DCE analysis. Future analysis will include also higher order radiomics features, such as textures, etc. The T2 relaxation times were not investigated as the clinical mpMRI protocol [30] did not include pulse sequence for T2 quantification. In the analysis of this small cohort, we included only first order radiomic features to avoid overfitting. Although ADT is known to cause significant changes in and to ADC and K^trans^ values in both tumorous and benign prostatic tissue [53] because of the low patients’ numbers, in this study we did not consider ADT as a confounding factor. In a preliminary study [54], we did not find measurable differences in the imaging variables post-RT, indicating that the magnitude of the radiation-induced changes outweighs the possible ADT effects. However, this evaluation should be considered in a larger cohort. Similarly, in future work the clinical characteristics of the patients, such as age, PSA, prostate volume, Gleason Score, etc., will be added to the radiomics variables for the combined clinico-radiomics model. This work aimed at exploring the prognostic value of quantitative mpMRI features as a potential clinical tool to identify which patients would fail curative radiotherapy. As such, many questions for clinical post-radiotherapy management selection remain beyond the scope of this work.

## 5. Conclusions

In conclusion, our study demonstrated that RT causes significant changes to quantitative perfusion and diffusion imaging features in both tumorous and normally-appearing prostatic tissue. Because of the noninvasive nature of the mpMRI, acquiring multiple exams post-RT are feasible to monitor treatment response. Several quantitative imaging features are promising predictors of treatment failure based on post-treatment positive biopsy, a strong marker for clinically relevant endpoints. By creating a pipeline for automatic analysis of longitudinal mpMRI datasets, these studies can be expanded to larger patient cohorts for straightening, conformation, and validation of these preliminary results.

## Figures and Tables

**Table 1:** Radiomics variables and the abbreviations used in radiomics variables name-convention *.

ROI	ADC	DCE
Gross Tumor Volume (GTV) Peritumoral GTV in PZ zone (PT-PZ) Peritumoral GTV in TZ zone (PT-TZ) Normally-Appearing Peripheral Zone (NAT-PZ) Normally-Appearing Transition Zone (NAT-TZ)	10% 25% 50% 75% 90% Mean Standard deviation (SD) Kurtosis (Kurt) Skewness (Skew)	K^trans^ k_ep_ v_e_ AUC90 AUC120 t_onset_

Abbreviations: ROI = Region of Interest, ADC = Apparent Diffusion Coefficient; DCE = Dynamic Contrast Enhanced MRI. * Variable names are concatenation of ROI, image sequence and radiomics feature. For example, the GTV ADC 90% will be GTV_ADC_90. Similarly, the same feature in NAT-TZ will be NAT-TZ_ADC_90.

**Table 2:** Clinical and mpMRI exams characteristics of the patient cohort.

	N (%)
**Age, years (mean ± stdev)**	68 ± 8
**Ethnicity**	
**Hispanic**	8 (32%)
**Non-Hispanic**	17 (68%)
**PSA, ng/mL (mean ± stdev)**	7.54 ± 3.51
**Grade Group**	
**GG1**	11 (44%)
**GG2**	6 (24%)
**GG3**	4 (16%)
**GG4**	3 (12%)
**GG5**	1 (4%)
**T-category**	
**T1c**	16 (64%)
**T2a**	5 (20%)
**T2b**	3 (12%)
**T2c**	1 (4%)
**Number of GTVs**	
**1**	12 (48%)
**2**	11 (44%)
**3**	2 (8%)
**Zonal location of GTVs**	
**PZ**	34 (85%)
**TZ**	3 (7.5%)
**PZ/TZ**	3 (7.5%)
**Number of post-RT exams**	
**2**	6 (24%)
**3**	19 (76%)
**Total MRI exams**	94
**MRI scanner**	
**Discovery**	64 (68%)
**Skyra**	24 (26%)
**Symphony**	1 (1%)
**TrioTim**	5 (5%)

**Table 3:** Quantitative imaging features extracted from longitudinal mpMRI exams before and following LEAD radiotherapy.

			GTV	NAT-PZ	NAT-TZ
Sequence	Feature	MRI Scan	Mean ± Stdev	Mean ± Stdev	Mean ± Stdev
DWI	ADC (mm^2^/sec)	Baseline	1177.84 ± 217.12	1616.37 ± 304.02	1381.76 ± 170.34
		3 months post-RT	1362.45 ± 191.93	1435.44 ± 290.54	1303.92 ± 226.71
		9 months post-RT	1474.76 ± 241.12	1493.89 ± 217.73	1357.37 ± 171.65
		24 months post-RT	1388.68 ± 212.14	1445.18 ± 206.83	1361.00 ± 251.03
DCE	K^trans^ (min^−1^)	Baseline	0.12 ± 0.05	0.08 ± 0.05	0.12 ± 0.06
		3 months post-RT	0.12 ± 0.07	0.13 ± 0.05	0.14 ± 0.07
		9 months post-RT	0.11 ± 0.05	0.09 ± 0.04	0.09 ± 0.05
		24 months post-RT	0.09 ± 0.05	0.10 ± 0.04	0.12 ± 0.05
	k_ep_ (min^−1^)	Baseline	0.49 ± 0.15	0.37 ± 0.19	0.40 ± 0.29
		3 months post-RT	0.25 ± 0.13	0.27 ± 0.13	0.30 ± 0.12
		9 months post-RT	0.24 ± 0.12	0.20 ± 0.13	0.21 ± 0.13
		24 months post-RT	0.16 ± 0.09	0.17 ± 0.08	0.22 ± 0.07
	v_e_ (%)	Baseline	25.54 ± 8.21	22.82 ± 11.12	33.55 ± 12.73
		3 months post-RT	58.80 ± 34.19	55.13 ± 25.12	52.66 ± 25.03
		9 months post-RT	46.37 ± 18.85	66.34 ± 60.06	46.62 ± 17.72
		24 months post-RT	66.58 ± 37.17	65.15 ± 31.30	59.30 ± 22.60

## Data Availability

Research data is stored in an institutional repository and will be shared upon request to the corresponding author.

## Appendix A

**Table A1:** MRI acquisition parameters for mpMRI sequences.

Scanner	Parameters	T2W	DWI	DCE
**GE-Discovery**	Pulse Sequence	FSE	EPI	SPRG
	TR (ms)	10763	9500	4.052
	TE (ms)	104.944	52.6	1.78
	Pixel size (mm)	1.25 × 1.25 × 2.5	1.25 × 1.25 × 2.5	1.25 × 1.25 × 2.5
	Matrix	256 × 256 × 72	256 × 256 × 36	256 × 256 × 72
	b-values		50–500–1000	
	DCE-MRI Temporal resolution (sec)			27–36
**Siemens-Skyra**	Pulse Sequence	FSE	EPI	GR SP
	TR (ms)	6100	6600	5.24
	TE (ms)	114	91	2.33
	Pixel size (mm)	0.7 × 0.7 × 2.5	2.93 × 2.93 × 2.5	0.7 × 0.7 × 2.5
	Matrix	512 × 384 × 72	128 × 96 × 38	512 × 384 × 72
	b-values		50–500–1400	
	DCE-MRI Temporal resolution (sec)			30–35

Abbreviations: T2W = T2-weighted MRI; DWI = Diffusion-Weighted Imaging; DCE = Dynamic Contrast-Enhanced MRI.

**Table A2:** Comparison of quantitative imaging features at baseline. The *p*-values from the Student’s t-test between ROIs are reported. Statistically significant values (<0.05) are marked with *.

Feature	GTV	NAT-PZ	NAT-TZ
**ADC**			
**GTV**	0		
**NAT-PZ**	<0.0001 *	0	
**NAT-TZ**	0.002 *	0.004 *	0
**K^trans^**			
**GTV**	0		
**NAT-PZ**	0.003 *	0	
**NAT-TZ**	0.832	0.017 *	0
**k_ep_**			
**GTV**	0		
**NAT-PZ**	0.025 *	0	
**NAT-TZ**	0.233	0.631	0
**v_e_**			
**GTV**	0		
**NAT-PZ**	0.372	0	
**NAT-TZ**	0.020 *	0.006 *	0

**Table A3:** Comparison of quantitative imaging features at baseline. The *p*-values from Student’s t-test between ROIs are reported. Statistically significant values (<0.05) are marked with *.

Features	GTV	NAT-PZ	NAT-TZ
**ADC**			
**S21**	0.010 *	0.001 *	0.825
**S31**	0.001 *	0.347	0.674
**S32**	0.106	0.010 *	0.402
**S41**	0.012 *	0.197	0.699
**S42**	0.696	0.039 *	0.474
**S43**	0.270	0.643	0.959
**K^trans^**			
**S21**	0.946	0.292	0.222
**S31**	0.285	0.559	0.092
**S32**	0.346	0.472	0.005 *
**S41**	0.033 *	0.350	0.939
**S42**	0.075	0.910	0.246
**S43**	0.306	0.500	0.055
**k_ep_**			
**S21**	<0.001 *	0.060	0.126
**S31**	<0.001 *	0.004 *	0.013 *
**S32**	0.734	0.133	0.038 *
**S41**	<0.001 *	0.001 *	0.017 *
**S42**	0.020 *	0.014 *	0.026 *
**S43**	0.042 *	0.382	0.882
**v_e_**			
**S21**	<0.001 *	<0.001 *	0.003 *
**S31**	<0.001 *	0.002 *	0.011 *
**S32**	0.178	0.440	0.398
**S41**	<0.001 *	<0.001 *	<0.001 *
**S42**	0.513	0.287	0.410
**S43**	0.050	0.944	0.076
